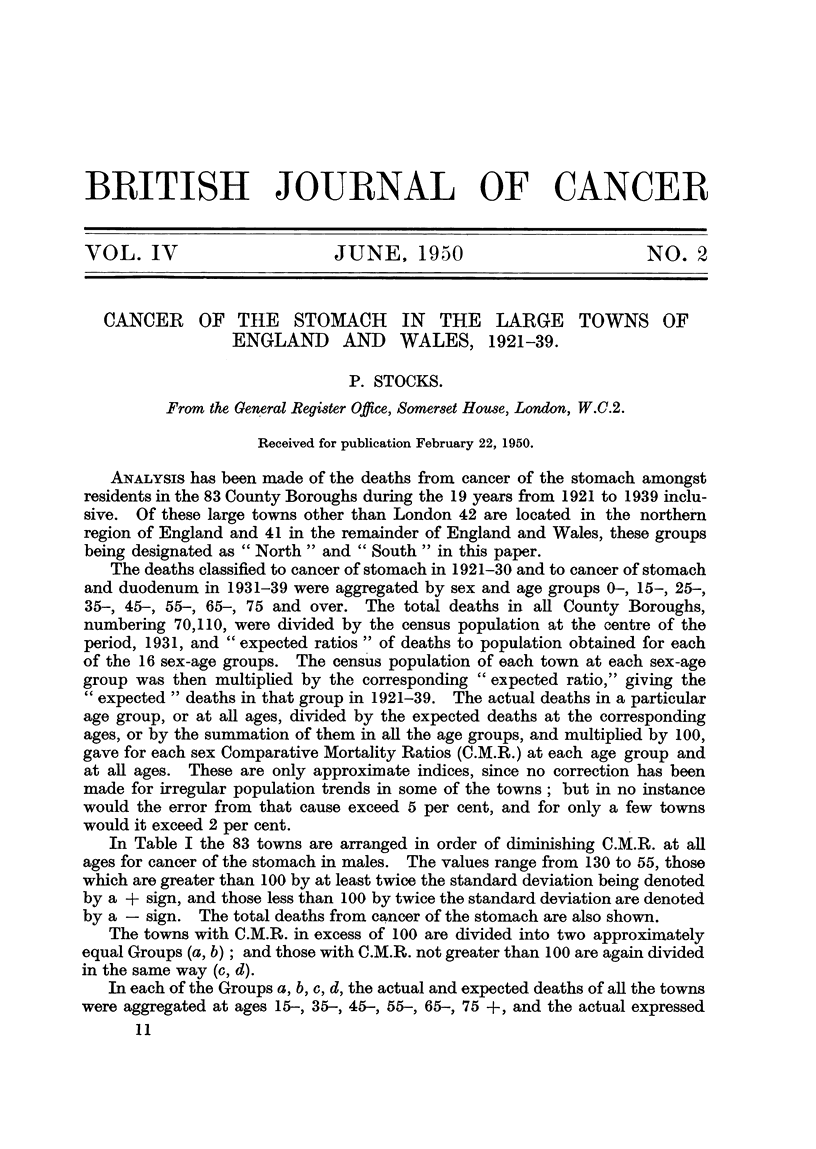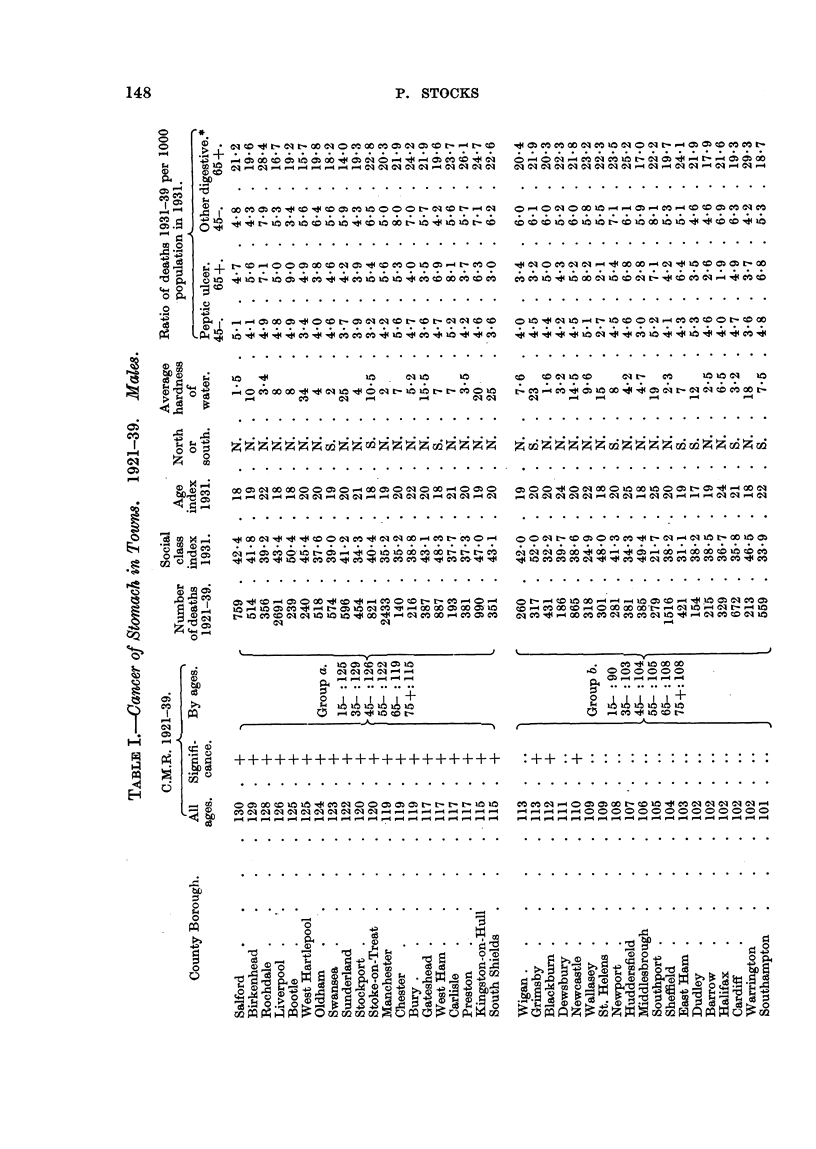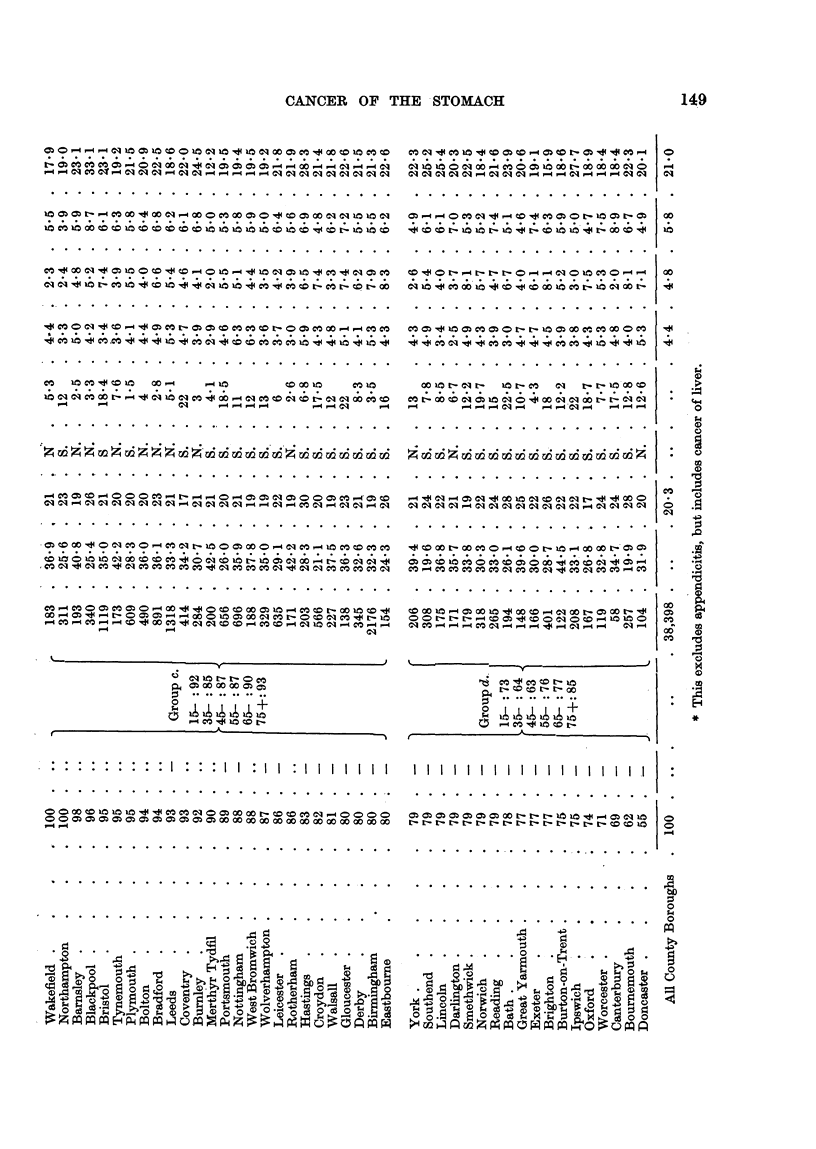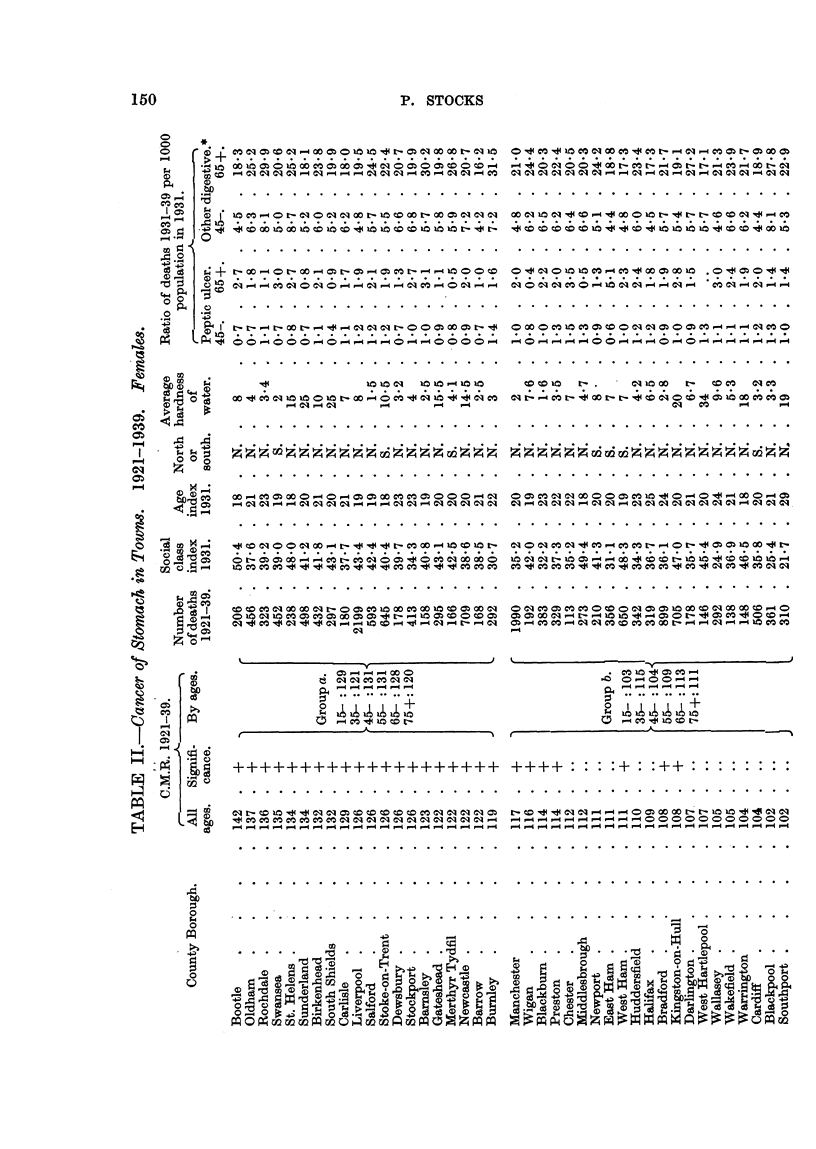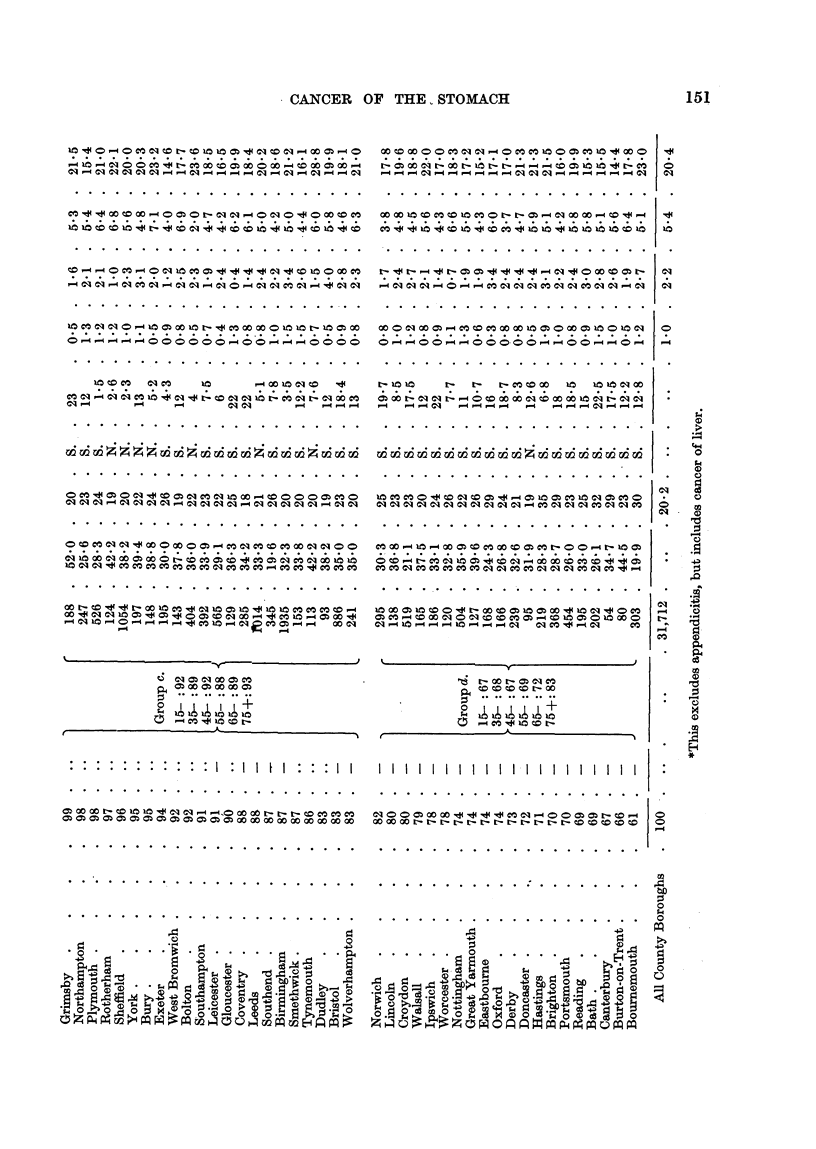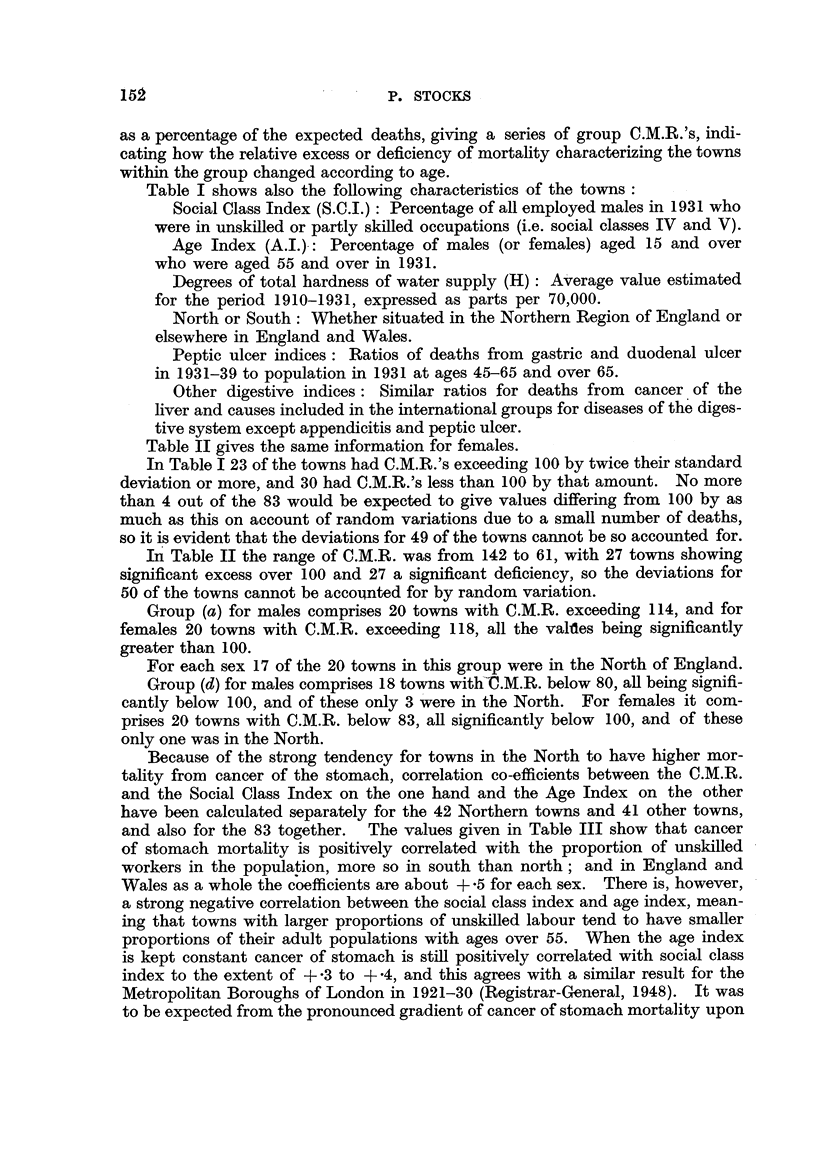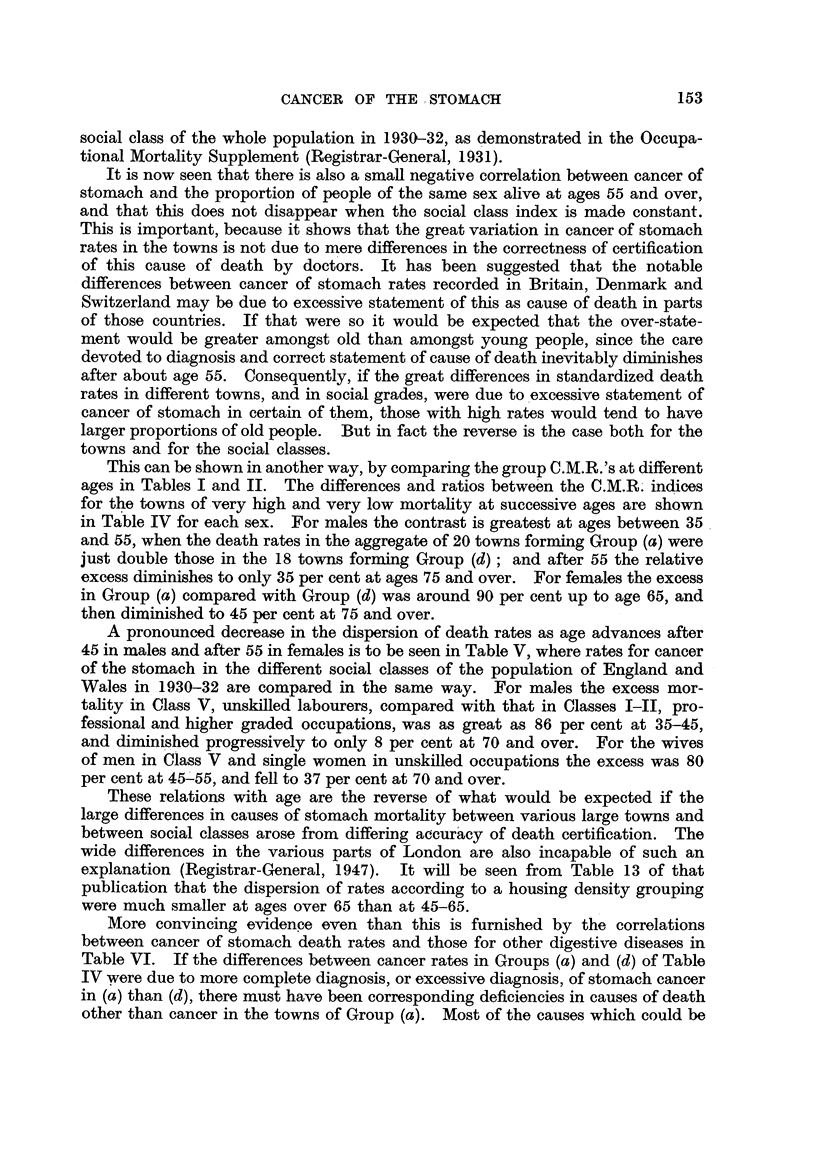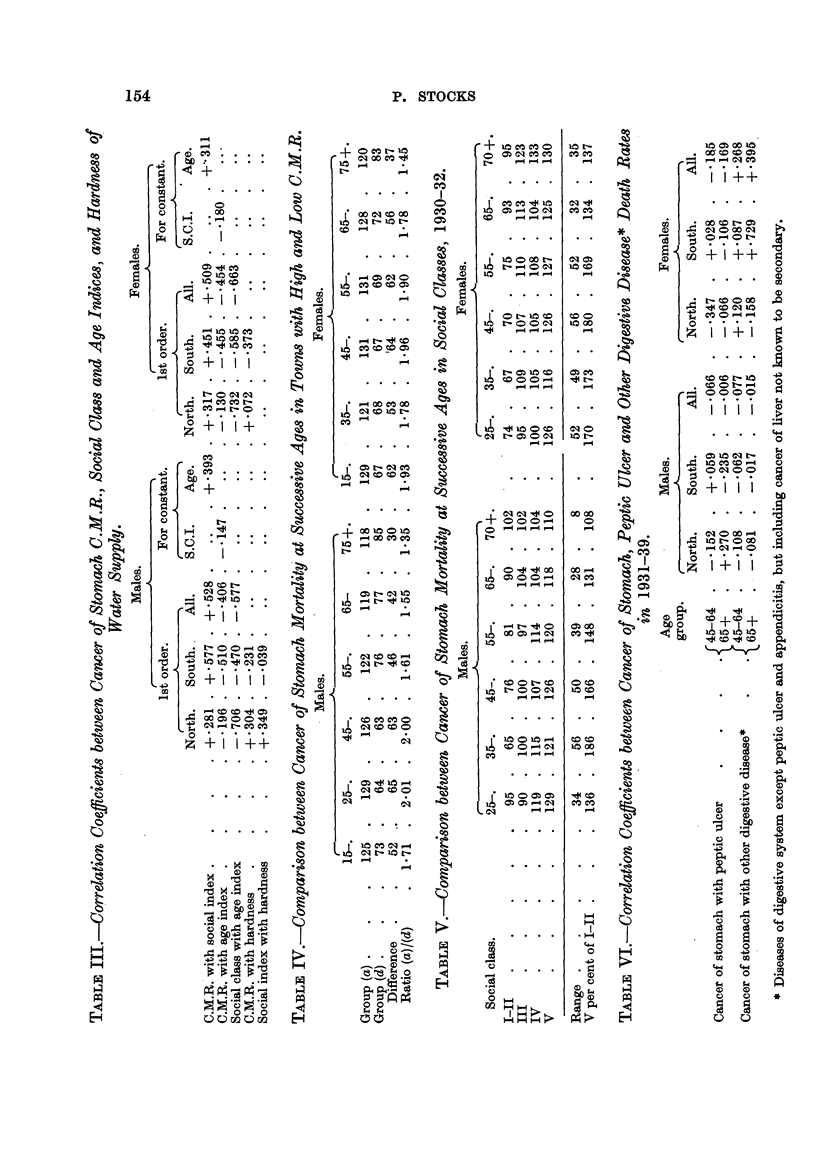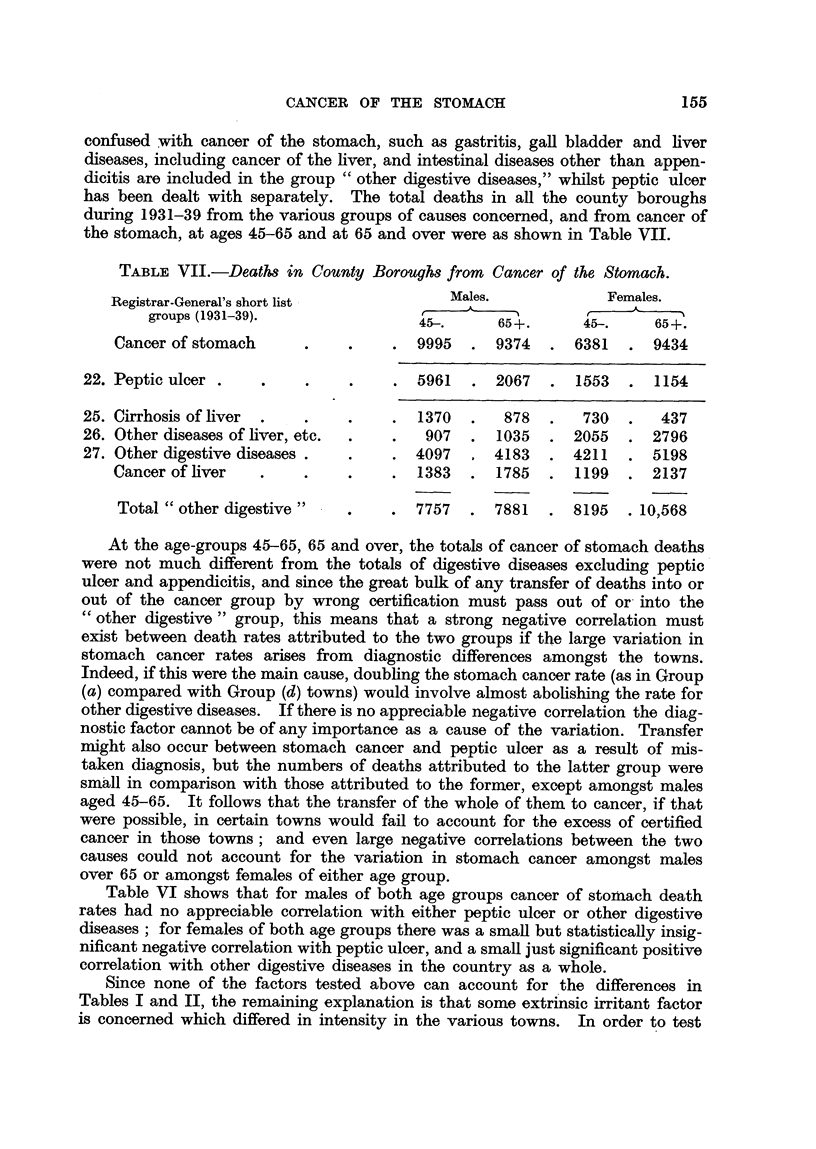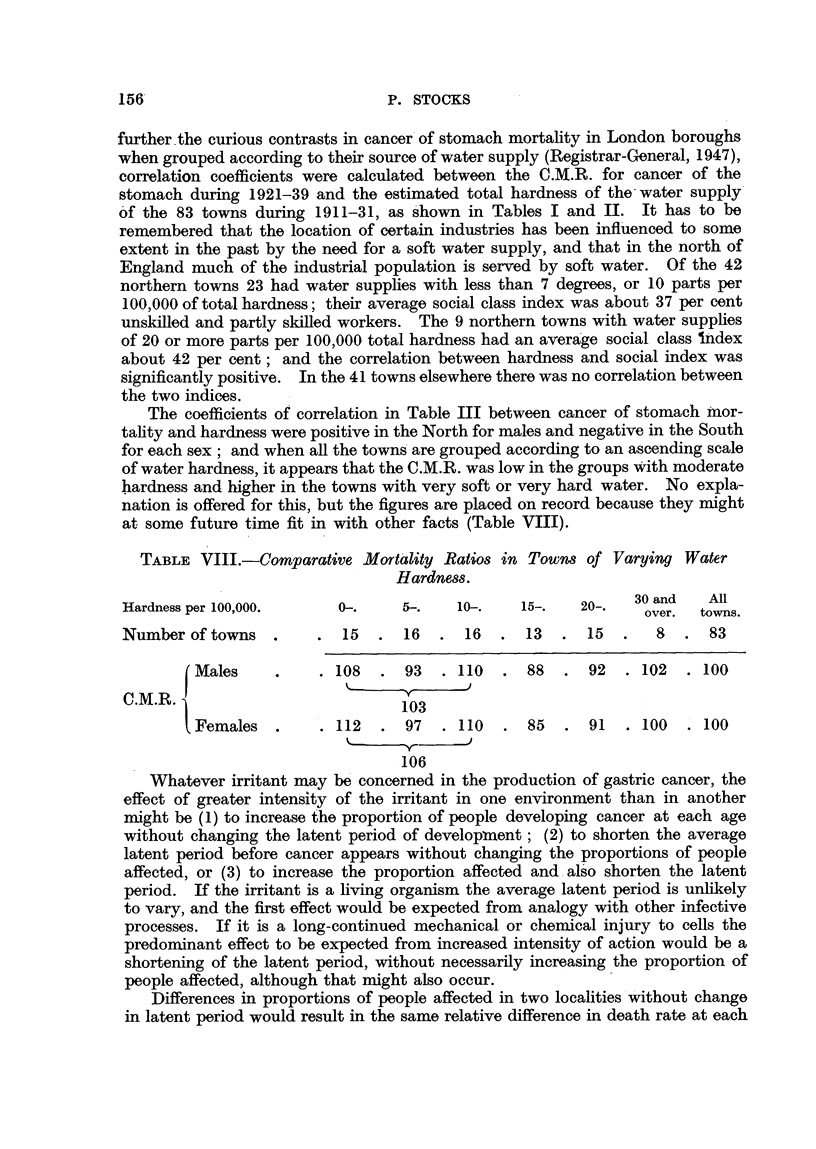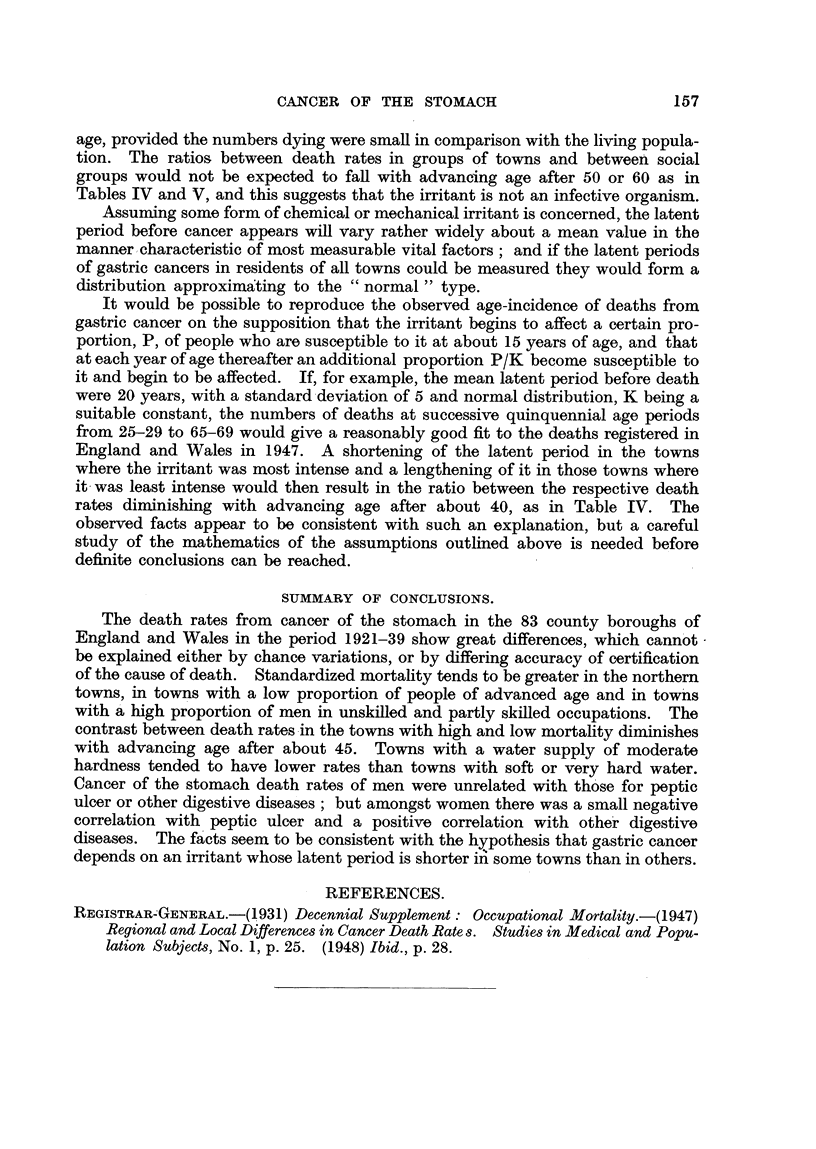# Cancer of the Stomach in the Large Towns of England and Wales, 1921-39

**DOI:** 10.1038/bjc.1950.15

**Published:** 1950-06

**Authors:** P. Stocks


### BRITISH JOURNAL OF CANCER

VOL. IV JUNE, 1950 NO. 2

### CANCER OF THE STOMACH IN THE LARGE TOWNS OF ENGLAND AND WALES, 1921-39.

P. STOCKS.

*From the General Register Office, Somerset House, London, W.C.2.*

Received for publication February 22, 1950.

ANALYSIS has been made of the deaths from cancer of the stomach amongst residents in the 83 County Boroughs during the 19 years from 1921 to 1939 inclusive. Of these large towns other than London 42 are located in the northern region of England and 41 in the remainder of England and Wales, these groups being designated as " North " and " South " in this paper.

The deaths classified to cancer of stomach in 1921–30 and to cancer of stomach and duodenum in 1931–39 were aggregated by sex and age groups 0–, 15–, 25–, 35–, 45–, 55–, 65–, 75 and over. The total deaths in all County Boroughs, numbering 70,110, were divided by the census population at the centre of the period, 1931, and " expected ratios " of deaths to population obtained for each of the 16 sex-age groups. The census population of each town at each sex-age group was then multiplied by the corresponding " expected ratio," giving the " expected " deaths in that group in 1921–39. The actual deaths in a particular age group, or at all ages, divided by the expected deaths at the corresponding ages, or by the summation of them in all the age groups, and multiplied by 100, gave for each sex Comparative Mortality Ratios (C.M.R.) at each age group and at all ages. These are only approximate indices, since no correction has been made for irregular population trends in some of the towns ; but in no instance would the error from that cause exceed 5 per cent, and for only a few towns would it exceed 2 per cent.

In Table I the 83 towns are arranged in order of diminishing C.M.R. at all ages for cancer of the stomach in males. The values range from 130 to 55, those which are greater than 100 by at least twice the standard deviation being denoted by a + sign, and those less than 100 by twice the standard deviation are denoted by a − sign. The total deaths from cancer of the stomach are also shown.

The towns with C.M.R. in excess of 100 are divided into two approximately equal Groups (*a*, *b*) ; and those with C.M.R. not greater than 100 are again divided in the same way (*c*, *d*).

In each of the Groups *a*, *b*, *c*, *d*, the actual and expected deaths of all the towns were aggregated at ages 15–, 35–, 45–, 55–, 65–, 75 +, and the actual expressed

TABLE I.—*Cancer of Stomach in Towns.* 1921–39. *Males.*

| County Borough. | C.M.R. 1921–39. All ages. | C.M.R. 1921–39. Significance. | C.M.R. 1921–39. By ages. | Number of deaths 1921–39. | Social class index 1931. | Age index 1931. | North or south. | Average hardness of water. | Ratio of deaths 1931–39 per 1000 population in 1931. Peptic ulcer. 45–. | Peptic ulcer. 65+. | Other digestive.* 45–. | Other digestive.* 65+. |
|---|---|---|---|---|---|---|---|---|---|---|---|---|
| Salford | 130 | + | | 759 | 42·4 | 18 | N. | 1·5 | 5·1 | 4·7 | 4·8 | 21·2 |
| Birkenhead | 129 | + | | 514 | 41·8 | 19 | N. | 10 | 4·1 | 5·6 | 4·3 | 19·6 |
| Rochdale | 128 | + | | 356 | 39·2 | 22 | N. | 3·4 | 4·9 | 7·1 | 7·9 | 28·4 |
| Liverpool | 126 | + | | 2691 | 43·4 | 18 | N. | 8 | 4·8 | 5·0 | 5·3 | 16·7 |
| Bootle | 125 | + | | 239 | 50·4 | 18 | N. | 8 | 4·9 | 9·0 | 3·4 | 19·2 |
| West Hartlepool | 125 | + | | 240 | 45·4 | 20 | N. | 34 | 3·4 | 4·9 | 5·6 | 15·7 |
| Oldham | 124 | + | Group *a*. | 518 | 37·6 | 20 | N. | 4 | 4·0 | 3·8 | 6·4 | 19·8 |
| Swansea | 123 | + | | 574 | 39·0 | 19 | S. | 2 | 4·6 | 4·6 | 5·6 | 18·2 |
| Sunderland | 122 | + | 15– : 125 | 596 | 41·2 | 20 | N. | 25 | 3·7 | 4·2 | 5·9 | 14·0 |
| Stockport | 120 | + | 35– : 129 | 454 | 34·3 | 21 | N. | 4 | 3·9 | 3·9 | 4·3 | 19·3 |
| Stoke-on-Treat | 120 | + | 45– : 126 | 821 | 40·4 | 18 | S. | 10·5 | 3·2 | 5·4 | 6·5 | 22·8 |
| Manchester | 119 | + | 55– : 122 | 2433 | 35·2 | 19 | N. | 2 | 4·2 | 5·6 | 5·0 | 20·3 |
| Chester | 119 | + | 65– : 119 | 140 | 35·2 | 20 | N. | 7 | 5·6 | 5·3 | 8·0 | 21·9 |
| Bury | 119 | + | 75+: 115 | 216 | 38·8 | 22 | N. | 5·2 | 4·7 | 4·0 | 7·0 | 24·2 |
| Gateshead | 117 | + | | 387 | 43·1 | 20 | N. | 15·5 | 3·6 | 3·5 | 5·7 | 21·9 |
| West Ham | 117 | + | | 887 | 48·3 | 18 | S. | 7 | 4·7 | 6·9 | 4·2 | 19·6 |
| Carlisle | 117 | + | | 193 | 37·7 | 21 | N. | 7 | 5·2 | 8·1 | 5·6 | 23·7 |
| Preston | 117 | + | | 381 | 37·3 | 20 | N. | 3·5 | 4·2 | 3·7 | 5·7 | 26·1 |
| Kingston-on-Hull | 115 | + | | 990 | 47·0 | 19 | N. | 20 | 4·6 | 6·3 | 7·1 | 24·7 |
| South Shields | 115 | + | | 351 | 43·1 | 20 | N. | 25 | 3·6 | 3·0 | 6·2 | 22·6 |
| Wigan | 113 | .. | | 260 | 42·0 | 19 | N. | 7·6 | 4·0 | 3·4 | 6·0 | 20·4 |
| Grimsby | 113 | + | | 317 | 52·0 | 20 | S. | 23 | 4·5 | 3·2 | 6·1 | 21·9 |
| Blackburn | 112 | + | | 431 | 32·2 | 20 | N. | 1·6 | 4·4 | 5·0 | 6·0 | 20·3 |
| Dewsbury | 111 | .. | | 186 | 39·7 | 24 | N. | 3·2 | 4·2 | 4·3 | 5·2 | 22·3 |
| Newcastle | 110 | + | | 865 | 38·6 | 20 | N. | 14·5 | 4·5 | 5·2 | 6·0 | 21·8 |
| Wallasey | 109 | .. | Group *b*. | 318 | 24·9 | 22 | N. | 9·6 | 5·1 | 8·2 | 5·8 | 23·2 |
| St. Helens | 109 | .. | | 301 | 48·0 | 18 | N. | 15 | 2·7 | 2·1 | 5·5 | 22·3 |
| Newport | 108 | .. | 15– : 90 | 281 | 41·3 | 20 | S. | 8 | 4·5 | 5·4 | 7·1 | 23·5 |
| Huddersfield | 107 | .. | 35– : 103 | 381 | 34·3 | 25 | N. | 4·2 | 4·6 | 6·8 | 6·1 | 25·2 |
| Middlesbrough | 106 | .. | 45– : 104 | 385 | 49·4 | 18 | N. | 4·7 | 3·0 | 2·8 | 5·9 | 17·0 |
| Southport | 105 | .. | 55– : 105 | 279 | 21·7 | 25 | N. | 19 | 5·2 | 7·1 | 8·1 | 22·2 |
| Sheffield | 104 | .. | 65– : 108 | 1516 | 38·2 | 20 | N. | 2·3 | 4·1 | 4·2 | 5·3 | 19·7 |
| East Ham | 103 | .. | 75+: 108 | 421 | 31·1 | 19 | S. | 7 | 4·3 | 6·4 | 5·1 | 24·1 |
| Dudley | 102 | .. | | 154 | 38·2 | 17 | S. | 12 | 5·3 | 3·5 | 4·6 | 21·9 |
| Barrow | 102 | .. | | 215 | 38·5 | 19 | N. | 2·5 | 4·6 | 2·6 | 4·6 | 17·9 |
| Halifax | 102 | .. | | 329 | 36·7 | 24 | N. | 6·5 | 4·0 | 1·9 | 6·9 | 21·6 |
| Cardiff | 102 | .. | | 672 | 35·8 | 21 | S. | 3·2 | 4·7 | 4·9 | 6·3 | 19·3 |
| Warrington | 102 | .. | | 213 | 46·5 | 18 | N. | 18 | 3·6 | 3·7 | 4·2 | 29·3 |
| Southampton | 101 | .. | | 559 | 33·9 | 22 | S. | 7·5 | 4·8 | 6·8 | 5·3 | 18·7 |

| | | | | | | | | | | | | |
|---|---|---|---|---|---|---|---|---|---|---|---|---|
| Wakefield | 100 | .. | Group c.<br>15– : 92<br>35– : 85<br>45– : 87<br>55– : 87<br>65– : 90<br>75+: 93 | 183 | 36·9 | 21 | N. | 5·3 | 4·4 | 2·3 | 5·5 | 17·9 |
| Northampton | 100 | .. | | 311 | 25·6 | 23 | S. | 12 | 3·3 | 2·4 | 3·9 | 19·0 |
| Barnsley | 98 | .. | | 193 | 40·8 | 19 | N. | 2·5 | 5·0 | 4·8 | 5·9 | 23·1 |
| Blackpool | 96 | .. | | 340 | 25·4 | 26 | N. | 3·3 | 4·2 | 5·2 | 8·7 | 33·1 |
| Bristol | 95 | .. | | 1119 | 35·0 | 21 | S | 18·4 | 3·4 | 7·4 | 6·1 | 23·1 |
| Tynemouth | 95 | .. | | 173 | 42·2 | 20 | N. | 7·6 | 3·6 | 3·9 | 6·3 | 19·2 |
| Plymouth | 95 | .. | | 609 | 28·3 | 20 | S. | 1·5 | 4·1 | 5·5 | 5·8 | 21·5 |
| Bolton | 94 | .. | | 490 | 36·0 | 20 | N. | 4 | 4·4 | 4·0 | 6·4 | 20·9 |
| Bradford | 94 | .. | | 891 | 36·1 | 23 | N. | 2·8 | 4·9 | 6·6 | 6·8 | 22·5 |
| Leeds | 93 | — | | 1318 | 33·3 | 21 | N. | 5·1 | 5·3 | 5·4 | 6·2 | 18·6 |
| Coventry | 93 | .. | | 414 | 34·2 | 17 | S. | 22 | 4·7 | 4·6 | 6·1 | 22·0 |
| Burnley | 92 | .. | | 284 | 30·7 | 21 | N. | 3 | 3·9 | 4·1 | 6·8 | 24·5 |
| Merthyr Tydfil | 90 | .. | | 200 | 42·5 | 21 | S. | 4·1 | 2·9 | 2·0 | 5·0 | 12·2 |
| Portsmouth | 89 | — | | 656 | 26·0 | 20 | S. | 18·5 | 4·6 | 5·5 | 5·3 | 19·5 |
| Nottingham | 88 | — | | 696 | 35·9 | 21 | S. | 11 | 6·3 | 5·1 | 5·8 | 19·4 |
| West Bromwich | 88 | .. | | 188 | 37·8 | 19 | S. | 12 | 6·3 | 4·4 | 5·9 | 19·5 |
| Wolverhampton | 87 | — | | 329 | 35·0 | 19 | S. | 13 | 3·6 | 3·5 | 5·0 | 19·2 |
| Leicester | 86 | — | | 635 | 29·1 | 22 | S. | 6 | 3·7 | 4·2 | 6·4 | 21·8 |
| Rotherham | 86 | .. | | 171 | 42·2 | 19 | N. | 2·6 | 3·0 | 3·9 | 5·6 | 21·9 |
| Hastings | 83 | — | | 203 | 28·3 | 30 | S. | 6·8 | 5·9 | 6·5 | 6·9 | 28·3 |
| Croydon | 82 | — | | 566 | 21·1 | 20 | S. | 17·5 | 4·3 | 7·4 | 4·8 | 21·4 |
| Walsall | 81 | — | | 227 | 37·5 | 19 | S. | 12 | 4·8 | 3·3 | 6·2 | 21·8 |
| Gloucester | 80 | — | | 138 | 36·3 | 23 | S. | 22 | 5·1 | 7·4 | 7·2 | 22·6 |
| Derby | 80 | — | | 345 | 32·6 | 21 | S. | 8·3 | 4·1 | 6·2 | 5·5 | 21·5 |
| Birmingham | 80 | — | | 2176 | 32·3 | 19 | S. | 3·5 | 5·3 | 7·9 | 5·5 | 21·3 |
| Eastbourne | 80 | — | | 154 | 24·3 | 26 | S. | 16 | 4·3 | 8·3 | 6·2 | 22·6 |
| York | 79 | — | Group d.<br>15– : 73<br>35– : 64<br>45– : 63<br>55– : 76<br>65– : 77<br>75+: 85 | 206 | 39·4 | 21 | N. | 13 | 4·3 | 2·6 | 4·9 | 22·3 |
| Southend | 79 | — | | 308 | 19·6 | 24 | S. | 7·8 | 4·9 | 5·4 | 6·1 | 25·2 |
| Lincoln | 79 | — | | 175 | 36·8 | 22 | S. | 8·5 | 3·4 | 4·0 | 6·1 | 25·4 |
| Darlington | 79 | — | | 171 | 35·7 | 21 | N. | 6·7 | 2·5 | 3·7 | 7·0 | 20·3 |
| Smethwick | 79 | — | | 179 | 33·8 | 19 | S. | 12·2 | 4·9 | 8·1 | 5·3 | 22·5 |
| Norwich | 79 | — | | 318 | 30·3 | 22 | S. | 19·7 | 4·3 | 5·7 | 5·2 | 18·4 |
| Reading | 79 | — | | 265 | 33·0 | 24 | S. | 15 | 3·9 | 4·7 | 7·4 | 21·6 |
| Bath | 78 | — | | 194 | 26·1 | 28 | S. | 22·5 | 3·0 | 6·7 | 5·1 | 23·9 |
| Great Yarmouth | 77 | — | | 148 | 39·6 | 25 | S. | 10·7 | 4·7 | 4·0 | 4·6 | 20·6 |
| Exeter | 77 | — | | 166 | 30·0 | 22 | S. | 4·3 | 4·7 | 6·1 | 7·4 | 19·1 |
| Brighton | 77 | — | | 401 | 28·7 | 26 | S. | 18 | 4·5 | 8·1 | 6·3 | 15·9 |
| Burton-on-Trent | 75 | — | | 122 | 44·5 | 22 | S. | 12·2 | 3·9 | 5·2 | 5·9 | 18·6 |
| Ipswich | 75 | — | | 208 | 33·1 | 22 | S. | 22 | 3·8 | 3·0 | 5·0 | 27·7 |
| Oxford | 74 | — | | 167 | 26·8 | 17 | S. | 18·7 | 4·3 | 7·5 | 4·7 | 18·9 |
| Worcester | 71 | — | | 119 | 32·8 | 24 | S. | 7·7 | 5·3 | 5·3 | 7·5 | 18·4 |
| Canterbury | 69 | — | | 58 | 34·7 | 24 | S. | 17·5 | 4·8 | 2·0 | 8·9 | 18·4 |
| Bournemouth | 62 | — | | 257 | 19·9 | 28 | S. | 12·8 | 4·0 | 8·1 | 6·7 | 22·3 |
| Doncaster | 55 | — | | 104 | 31·9 | 20 | N. | 12·6 | 5·3 | 7·1 | 4·9 | 20·1 |
| All County Boroughs | 100 | .. | .. | 38,398 | .. | 20·3 | .. | .. | 4·4 | 4·8 | 5·8 | 21·0 |

* This excludes appendicitis, but includes cancer of liver.

### TABLE II.—*Cancer of Stomach in Towns.* 1921–1939. *Females.*

| County Borough. | C.M.R. 1921–39. All ages. | C.M.R. 1921–39. Significance. | C.M.R. 1921–39. By ages. | Number of deaths 1921–39. | Social class index 1931. | Age index 1931. | North or south. | Average hardness of water. | Ratio of deaths 1931–39 per 1000 population in 1931. Peptic ulcer. 45–. | Peptic ulcer. 65+. | Other digestive.* 45–. | Other digestive.* 65+. |
|---|---|---|---|---|---|---|---|---|---|---|---|---|
| Bootle | 142 | + | | 206 | 50·4 | 18 | N. | 8 | 0·7 | 2·7 | 4·5 | 18·3 |
| Oldham | 137 | + | | 456 | 37·6 | 21 | N. | 4 | 0·7 | 1·8 | 6·3 | 25·2 |
| Rochdale | 136 | + | | 323 | 39·2 | 23 | N. | 3·4 | 1·1 | 1·1 | 8·1 | 29·9 |
| Swansea | 135 | + | | 452 | 39·0 | 19 | S. | 2 | 0·7 | 3·0 | 5·0 | 20·6 |
| St. Helens | 134 | + | | 238 | 48·0 | 18 | N. | 15 | 0·8 | 2·7 | 8·7 | 25·2 |
| Sunderland | 134 | + | | 498 | 41·2 | 20 | N. | 25 | 0·7 | 0·8 | 5·2 | 18·1 |
| Birkenhead | 132 | + | Group *a.* | 432 | 41·8 | 21 | N. | 10 | 1·1 | 2·1 | 6·0 | 23·8 |
| South Shields | 132 | + | | 297 | 43·1 | 20 | N. | 25 | 0·4 | 0·9 | 5·2 | 19·9 |
| Carlisle | 129 | + | 15– : 129 | 180 | 37·7 | 21 | N. | 7 | 1·1 | 1·7 | 6·2 | 18·0 |
| Liverpool | 126 | + | 35– : 121 | 2199 | 43·4 | 19 | N. | 8 | 1·2 | 1·9 | 4·8 | 19·5 |
| Salford | 126 | + | 45– : 131 | 593 | 42·4 | 19 | N. | 1·5 | 1·2 | 2·1 | 5·7 | 24·5 |
| Stoke-on-Trent | 126 | + | 55– : 131 | 645 | 40·4 | 18 | S. | 10·5 | 1·2 | 1·9 | 5·5 | 22·4 |
| Dewsbury | 126 | + | 65– : 128 | 178 | 39·7 | 23 | N. | 3·2 | 0·7 | 1·3 | 6·6 | 20·7 |
| Stockport | 126 | + | 75+: 120 | 413 | 34·3 | 23 | N. | 4 | 1·0 | 2·7 | 6·8 | 19·9 |
| Barnsley | 123 | + | | 158 | 40·8 | 19 | N. | 2·5 | 1·0 | 3·1 | 5·7 | 30·2 |
| Gateshead | 122 | + | | 295 | 43·1 | 20 | N. | 15·5 | 0·9 | 1·1 | 5·8 | 19·8 |
| Merthyr Tydfil | 122 | + | | 166 | 42·5 | 20 | S. | 4·1 | 0·8 | 0·5 | 5·9 | 26·8 |
| Newcastle | 122 | + | | 709 | 38·6 | 20 | N. | 14·5 | 0·9 | 2·0 | 7·2 | 20·7 |
| Barrow | 122 | + | | 168 | 38·5 | 21 | N. | 2·5 | 0·7 | 1·0 | 4·2 | 16·2 |
| Burnley | 119 | + | | 292 | 30·7 | 22 | N. | 3 | 1·4 | 1·6 | 7·2 | 31·5 |
| Manchester | 117 | + | | 1990 | 35·2 | 20 | N. | 2 | 1·0 | 2·0 | 4·8 | 21·0 |
| Wigan | 116 | + | | 192 | 42·0 | 19 | N. | 7·6 | 0·8 | 0·4 | 6·2 | 24·4 |
| Blackburn | 114 | + | | 383 | 32·2 | 23 | N. | 1·6 | 1·0 | 2·2 | 6·5 | 20·3 |
| Preston | 114 | + | | 329 | 37·3 | 22 | N. | 3·5 | 1·3 | 2·0 | 6·2 | 22·4 |
| Chester | 112 | .. | | 113 | 35·2 | 22 | N. | 7 | 1·5 | 3·5 | 6·4 | 20·5 |
| Middlesbrough | 112 | .. | | 273 | 49·4 | 18 | N. | 4·7 | 1·3 | 0·5 | 6·6 | 20·3 |
| Newport | 111 | .. | | 210 | 41·3 | 20 | S. | 8 | 0·9 | 1·3 | 5·1 | 24·2 |
| East Ham | 111 | .. | Group *b.* | 356 | 31·1 | 20 | S. | 7 | 0·6 | 5·1 | 4·4 | 18·8 |
| West Ham | 111 | + | 15– : 103 | 650 | 48·3 | 19 | S. | 7 | 1·0 | 2·3 | 4·8 | 17·3 |
| Huddersfield | 110 | .. | 35– : 115 | 342 | 34·3 | 23 | N. | 4·2 | 1·2 | 2·4 | 6·0 | 23·4 |
| Halifax | 109 | .. | 45– : 104 | 319 | 36·7 | 25 | N. | 6·5 | 1·2 | 1·8 | 4·5 | 17·3 |
| Bradford | 108 | + | 55– : 109 | 899 | 36·1 | 24 | N. | 2·8 | 0·9 | 1·9 | 5·7 | 21·7 |
| Kingston-on-Hull | 108 | + | 65– : 113 | 705 | 47·0 | 20 | N. | 20 | 1·0 | 2·8 | 5·4 | 19·1 |
| Darlington | 107 | .. | 75+: 111 | 178 | 35·7 | 21 | N. | 6·7 | 0·9 | 1·5 | 5·7 | 27·2 |
| West Hartlepool | 107 | .. | | 146 | 45·4 | 20 | N. | 34 | 1·3 | .. | 5·7 | 17·1 |
| Wallasey | 105 | .. | | 292 | 24·9 | 24 | N. | 9·6 | 1·1 | 3·0 | 4·6 | 21·3 |
| Wakefield | 105 | .. | | 138 | 36·9 | 21 | N. | 5·3 | 1·1 | 2·4 | 6·6 | 23·9 |
| Warrington | 104 | .. | | 148 | 46·5 | 18 | N. | 18 | 1·1 | 1·9 | 6·2 | 21·7 |
| Cardiff | 104 | .. | | 506 | 35·8 | 20 | S. | 3·2 | 1·2 | 2·0 | 4·4 | 18·9 |
| Blackpool | 102 | .. | | 361 | 25·4 | 21 | N. | 3·3 | 1·3 | 1·4 | 8·1 | 27·8 |
| Southport | 102 | .. | | 310 | 21·7 | 29 | N. | 19 | 1·0 | 1·4 | 5·3 | 22·9 |

| | | | | | | | | | | | | |
|---|---|---|---|---|---|---|---|---|---|---|---|---|
| Grimsby | 99 | .. | | 188 | 52·0 | 20 | S. | 23 | 0·5 | 1·6 | 5·3 | 21·5 |
| Northampton | 98 | .. | | 247 | 25·6 | 23 | S. | 12 | 1·3 | 2·1 | 5·4 | 15·4 |
| Plymouth | 98 | .. | | 526 | 28·3 | 24 | S. | 1·5 | 1·2 | 2·1 | 6·4 | 21·0 |
| Rotherham | 97 | .. | | 124 | 42·2 | 19 | N. | 2·6 | 1·2 | 1·0 | 6·8 | 22·1 |
| Sheffield | 96 | .. | | 1054 | 38·2 | 20 | N. | 2·3 | 1·0 | 2·3 | 5·6 | 20·0 |
| York | 95 | .. | | 197 | 39·4 | 22 | N. | 13 | 1·1 | 3·1 | 4·8 | 20·3 |
| Bury | 95 | .. | | 148 | 38·8 | 24 | N. | 5·2 | 0·5 | 2·0 | 7·1 | 23·2 |
| Exeter | 94 | .. | Group *c.* | 195 | 30·0 | 26 | S. | 4·3 | 0·9 | 1·2 | 4·0 | 14·6 |
| West Bromwich | 92 | .. | 15– : 92 | 143 | 37·8 | 19 | S. | 12 | 0·8 | 2·5 | 6·9 | 17·7 |
| Bolton | 92 | .. | 35– : 89 | 404 | 36·0 | 22 | N. | 4 | 0·5 | 2·3 | 2·0 | 23·6 |
| Southampton | 91 | .. | 45– : 92 | 392 | 33·9 | 23 | S. | 7·5 | 0·7 | 1·9 | 4·7 | 18·5 |
| Leicester | 91 | — | 55– : 88 | 565 | 29·1 | 22 | S. | 6 | 0·4 | 2·4 | 4·2 | 16·5 |
| Gloucester | 90 | .. | 65– : 89 | 129 | 36·3 | 25 | S. | 22 | 1·3 | 0·4 | 6·2 | 19·9 |
| Coventry | 88 | — | 75+ : 93 | 285 | 34·2 | 18 | S. | 22 | 0·8 | 1·4 | 6·1 | 18·4 |
| Leeds | 88 | — | | 1014 | 33·3 | 21 | N. | 5·1 | 0·8 | 2·4 | 5·0 | 20·2 |
| Southend | 87 | — | | 345 | 19·6 | 26 | S. | 7·8 | 1·0 | 2·2 | 4·2 | 18·6 |
| Birmingham | 87 | — | | 1935 | 32·3 | 20 | S. | 3·5 | 1·5 | 3·4 | 5·0 | 21·2 |
| Smethwick | 87 | .. | | 153 | 33·8 | 20 | S. | 12·2 | 1·5 | 2·6 | 4·4 | 16·1 |
| Tynemouth | 86 | .. | | 113 | 42·2 | 20 | N. | 7·6 | 0·7 | 1·5 | 6·0 | 28·8 |
| Dudley | 83 | .. | | 93 | 38·2 | 19 | S. | 12 | 0·5 | 4·0 | 5·8 | 19·9 |
| Bristol | 83 | — | | 886 | 35·0 | 23 | S. | 18·4 | 0·9 | 2·8 | 4·6 | 18·1 |
| Wolverhampton | 83 | — | | 241 | 35·0 | 20 | S. | 13 | 0·8 | 2·3 | 6·3 | 21·0 |
| Norwich | 82 | — | | 295 | 30·3 | 25 | S. | 19·7 | 0·8 | 1·7 | 3·8 | 17·8 |
| Lincoln | 80 | — | | 138 | 36·8 | 23 | S. | 8·5 | 1·0 | 2·4 | 4·8 | 19·6 |
| Croydon | 80 | — | | 519 | 21·1 | 23 | S. | 17·5 | 1·2 | 2·7 | 4·5 | 18·8 |
| Walsall | 79 | — | | 165 | 37·5 | 20 | S. | 12 | 0·8 | 2·1 | 5·6 | 22·0 |
| Ipswich | 78 | — | | 186 | 33·1 | 24 | S. | 22 | 0·9 | 1·4 | 4·3 | 17·0 |
| Worcester | 78 | — | | 120 | 32·8 | 26 | S. | 7·7 | 1·1 | 0·7 | 6·6 | 18·3 |
| Nottingham | 74 | — | Group *d.* | 504 | 35·9 | 22 | S. | 11 | 1·3 | 1·9 | 5·5 | 17·2 |
| Great Yarmouth | 74 | — | | 127 | 39·6 | 26 | S. | 10·7 | 0·6 | 1·9 | 4·3 | 15·2 |
| Eastbourne | 74 | — | 15– : 67 | 168 | 24·3 | 29 | S. | 16 | 0·3 | 3·4 | 6·0 | 17·1 |
| Oxford | 74 | — | 35– : 68 | 166 | 26·8 | 24 | S. | 18·7 | 0·8 | 2·4 | 3·7 | 17·0 |
| Derby | 73 | — | 45– : 67 | 239 | 32·6 | 21 | S. | 8·3 | 0·8 | 2·4 | 4·7 | 21·3 |
| Doncaster | 72 | — | 55– : 69 | 95 | 31·9 | 19 | N. | 12·6 | 0·5 | 2·4 | 5·9 | 21·3 |
| Hastings | 71 | — | 65– : 72 | 219 | 28·3 | 35 | S. | 6·8 | 1·9 | 3·1 | 5·1 | 21·5 |
| Brighton | 70 | — | 75+ : 83 | 368 | 28·7 | 29 | S. | 18 | 1·0 | 2·2 | 4·2 | 16·0 |
| Portsmouth | 70 | — | | 454 | 26·0 | 23 | S. | 18·5 | 0·8 | 2·4 | 5·8 | 19·9 |
| Reading | 69 | — | | 195 | 33·0 | 25 | S. | 15 | 0·9 | 3·0 | 5·8 | 15·3 |
| Bath | 69 | — | | 202 | 26·1 | 32 | S. | 22·5 | 1·5 | 2·8 | 5·1 | 15·5 |
| Canterbury | 67 | — | | 54 | 34·7 | 29 | S. | 17·5 | 1·0 | 2·6 | 5·6 | 14·4 |
| Burton-on-Trent | 66 | — | | 80 | 44·5 | 23 | S. | 12·2 | 0·5 | 1·9 | 6·4 | 17·8 |
| Bournemouth | 61 | — | | 303 | 19·9 | 30 | S. | 12·8 | 1·2 | 2·7 | 5·1 | 23·0 |
| All County Boroughs | 100 | .. | .. | 31,712 | .. | 20·2 | .. | .. | 1·0 | 2·2 | 5·4 | 20·4 |

*This excludes appendicitis, but includes cancer of liver.

as a percentage of the expected deaths, giving a series of group C.M.R.'s, indicating how the relative excess or deficiency of mortality characterizing the towns within the group changed according to age.

Table I shows also the following characteristics of the towns :

Social Class Index (S.C.I.) : Percentage of all employed males in 1931 who were in unskilled or partly skilled occupations (i.e. social classes IV and V).

Age Index (A.I.) : Percentage of males (or females) aged 15 and over who were aged 55 and over in 1931.

Degrees of total hardness of water supply (H) : Average value estimated for the period 1910–1931, expressed as parts per 70,000.

North or South : Whether situated in the Northern Region of England or elsewhere in England and Wales.

Peptic ulcer indices : Ratios of deaths from gastric and duodenal ulcer in 1931–39 to population in 1931 at ages 45–65 and over 65.

Other digestive indices : Similar ratios for deaths from cancer of the liver and causes included in the international groups for diseases of the digestive system except appendicitis and peptic ulcer.

Table II gives the same information for females.

In Table I 23 of the towns had C.M.R.'s exceeding 100 by twice their standard deviation or more, and 30 had C.M.R.'s less than 100 by that amount. No more than 4 out of the 83 would be expected to give values differing from 100 by as much as this on account of random variations due to a small number of deaths, so it is evident that the deviations for 49 of the towns cannot be so accounted for.

In Table II the range of C.M.R. was from 142 to 61, with 27 towns showing significant excess over 100 and 27 a significant deficiency, so the deviations for 50 of the towns cannot be accounted for by random variation.

Group (*a*) for males comprises 20 towns with C.M.R. exceeding 114, and for females 20 towns with C.M.R. exceeding 118, all the values being significantly greater than 100.

For each sex 17 of the 20 towns in this group were in the North of England.

Group (*d*) for males comprises 18 towns with C.M.R. below 80, all being significantly below 100, and of these only 3 were in the North. For females it comprises 20 towns with C.M.R. below 83, all significantly below 100, and of these only one was in the North.

Because of the strong tendency for towns in the North to have higher mortality from cancer of the stomach, correlation co-efficients between the C.M.R. and the Social Class Index on the one hand and the Age Index on the other have been calculated separately for the 42 Northern towns and 41 other towns, and also for the 83 together. The values given in Table III show that cancer of stomach mortality is positively correlated with the proportion of unskilled workers in the population, more so in south than north ; and in England and Wales as a whole the coefficients are about +·5 for each sex. There is, however, a strong negative correlation between the social class index and age index, meaning that towns with larger proportions of unskilled labour tend to have smaller proportions of their adult populations with ages over 55. When the age index is kept constant cancer of stomach is still positively correlated with social class index to the extent of +·3 to +·4, and this agrees with a similar result for the Metropolitan Boroughs of London in 1921–30 (Registrar-General, 1948). It was to be expected from the pronounced gradient of cancer of stomach mortality upon social class of the whole population in 1930–32, as demonstrated in the Occupational Mortality Supplement (Registrar-General, 1931).

It is now seen that there is also a small negative correlation between cancer of stomach and the proportion of people of the same sex alive at ages 55 and over, and that this does not disappear when the social class index is made constant. This is important, because it shows that the great variation in cancer of stomach rates in the towns is not due to mere differences in the correctness of certification of this cause of death by doctors. It has been suggested that the notable differences between cancer of stomach rates recorded in Britain, Denmark and Switzerland may be due to excessive statement of this as cause of death in parts of those countries. If that were so it would be expected that the over-statement would be greater amongst old than amongst young people, since the care devoted to diagnosis and correct statement of cause of death inevitably diminishes after about age 55. Consequently, if the great differences in standardized death rates in different towns, and in social grades, were due to excessive statement of cancer of stomach in certain of them, those with high rates would tend to have larger proportions of old people. But in fact the reverse is the case both for the towns and for the social classes.

This can be shown in another way, by comparing the group C.M.R.'s at different ages in Tables I and II. The differences and ratios between the C.M.R. indices for the towns of very high and very low mortality at successive ages are shown in Table IV for each sex. For males the contrast is greatest at ages between 35 and 55, when the death rates in the aggregate of 20 towns forming Group (*a*) were just double those in the 18 towns forming Group (*d*); and after 55 the relative excess diminishes to only 35 per cent at ages 75 and over. For females the excess in Group (*a*) compared with Group (*d*) was around 90 per cent up to age 65, and then diminished to 45 per cent at 75 and over.

A pronounced decrease in the dispersion of death rates as age advances after 45 in males and after 55 in females is to be seen in Table V, where rates for cancer of the stomach in the different social classes of the population of England and Wales in 1930–32 are compared in the same way. For males the excess mortality in Class V, unskilled labourers, compared with that in Classes I–II, professional and higher graded occupations, was as great as 86 per cent at 35–45, and diminished progressively to only 8 per cent at 70 and over. For the wives of men in Class V and single women in unskilled occupations the excess was 80 per cent at 45–55, and fell to 37 per cent at 70 and over.

These relations with age are the reverse of what would be expected if the large differences in causes of stomach mortality between various large towns and between social classes arose from differing accuracy of death certification. The wide differences in the various parts of London are also incapable of such an explanation (Registrar-General, 1947). It will be seen from Table 13 of that publication that the dispersion of rates according to a housing density grouping were much smaller at ages over 65 than at 45–65.

More convincing evidence even than this is furnished by the correlations between cancer of stomach death rates and those for other digestive diseases in Table VI. If the differences between cancer rates in Groups (*a*) and (*d*) of Table IV were due to more complete diagnosis, or excessive diagnosis, of stomach cancer in (*a*) than (*d*), there must have been corresponding deficiencies in causes of death other than cancer in the towns of Group (*a*). Most of the causes which could be

TABLE III.—*Correlation Coefficients between Cancer of Stomach C.M.R., Social Class and Age Indices, and Hardness of Water Supply.*

| | Males. | | | | | Females. | | | | |
|---|---|---|---|---|---|---|---|---|---|---|
| | 1st order. | | | For constant. | | 1st order. | | | For constant. | |
| | North. | South. | All. | S.C.I. | Age. | North. | South. | All. | S.C.I. | Age. |
| C.M.R. with social index | +·281 | +·577 | +·528 | .. | +·393 | +·317 | +·451 | +·509 | .. | +·311 |
| C.M.R. with age index | −·196 | −·510 | −·406 | −·147 | .. | −·130 | −·455 | −·454 | −·180 | .. |
| Social class with age index | −·706 | −·470 | −·577 | .. | .. | −·732 | −·585 | −·663 | .. | .. |
| C.M.R. with hardness | +·304 | −·231 | .. | .. | .. | +·072 | −·373 | .. | .. | .. |
| Social index with hardness | +·349 | −·039 | .. | .. | .. | .. | .. | .. | .. | .. |

TABLE IV.—*Comparison between Cancer of Stomach Mortality at Successive Ages in Towns with High and Low C.M.R.*

| | Males. | | | | | | Females. | | | | | |
|---|---|---|---|---|---|---|---|---|---|---|---|---|
| | 15–. | 25–. | 45–. | 55–. | 65– | 75+. | 15–. | 35–. | 45–. | 55–. | 65–. | 75+. |
| Group (*a*) | 125 | 129 | 126 | 122 | 119 | 118 | 129 | 121 | 131 | 131 | 128 | 120 |
| Group (*d*) | 73 | 64 | 63 | 76 | 77 | 85 | 67 | 68 | 67 | 69 | 72 | 83 |
| Difference | 52 | 65 | 63 | 46 | 42 | 30 | 62 | 53 | 64 | 62 | 56 | 37 |
| Ratio (*a*)/(*d*) | 1·71 | 2·01 | 2·00 | 1·61 | 1·55 | 1·35 | 1·93 | 1·78 | 1·96 | 1·90 | 1·78 | 1·45 |

TABLE V.—*Comparison between Cancer of Stomach Mortality at Successive Ages in Social Classes,* 1930–32.

| Social class. | Males. | | | | | | Females. | | | | | |
|---|---|---|---|---|---|---|---|---|---|---|---|---|
| | 25–. | 35–. | 45–. | 55–. | 65–. | 70+. | 25–. | 35–. | 45–. | 55–. | 65–. | 70+. |
| I–II | 95 | 65 | 76 | 81 | 90 | 102 | 74 | 67 | 70 | 75 | 93 | 95 |
| III | 90 | 100 | 100 | 97 | 104 | 102 | 95 | 109 | 107 | 110 | 113 | 123 |
| IV | 119 | 115 | 107 | 114 | 104 | 104 | 100 | 105 | 105 | 108 | 104 | 133 |
| V | 129 | 121 | 126 | 120 | 118 | 110 | 126 | 116 | 126 | 127 | 125 | 130 |
| Range | 34 | 56 | 50 | 39 | 28 | 8 | 52 | 49 | 56 | 52 | 32 | 35 |
| V per cent of I–II | 136 | 186 | 166 | 148 | 131 | 108 | 170 | 173 | 180 | 169 | 134 | 137 |

TABLE VI.—*Correlation Coefficients between Cancer of Stomach, Peptic Ulcer and Other Digestive Disease* Death Rates in* 1931–39.

| | Age group. | Males. | | | Females. | | |
|---|---|---|---|---|---|---|---|
| | | North. | South. | All. | North. | South. | All. |
| Cancer of stomach with peptic ulcer | 45–64 | −·152 | +·059 | −·066 | −·347 | +·028 | −·185 |
| | 65+ | +·270 | −·235 | −·006 | −·066 | −·106 | −·169 |
| Cancer of stomach with other digestive disease* | 45–64 | −·108 | −·062 | −·077 | +·120 | +·087 | +·268 |
| | 65+ | −·081 | −·017 | −·015 | −·158 | +·729 | +·395 |

* Diseases of digestive system except peptic ulcer and appendicitis, but including cancer of liver not known to be secondary.

confused with cancer of the stomach, such as gastritis, gall bladder and liver diseases, including cancer of the liver, and intestinal diseases other than appendicitis are included in the group " other digestive diseases," whilst peptic ulcer has been dealt with separately. The total deaths in all the county boroughs during 1931–39 from the various groups of causes concerned, and from cancer of the stomach, at ages 45–65 and at 65 and over were as shown in Table VII.

TABLE VII.—*Deaths in County Boroughs from Cancer of the Stomach.*

| Registrar-General's short list groups (1931–39). | Males. | | Females. | |
|---|---|---|---|---|
| | 45–. | 65+. | 45–. | 65+. |
| Cancer of stomach | 9995 | 9374 | 6381 | 9434 |
| 22. Peptic ulcer | 5961 | 2067 | 1553 | 1154 |
| 25. Cirrhosis of liver | 1370 | 878 | 730 | 437 |
| 26. Other diseases of liver, etc. | 907 | 1035 | 2055 | 2796 |
| 27. Other digestive diseases | 4097 | 4183 | 4211 | 5198 |
| Cancer of liver | 1383 | 1785 | 1199 | 2137 |
| Total " other digestive " | 7757 | 7881 | 8195 | 10,568 |

At the age-groups 45–65, 65 and over, the totals of cancer of stomach deaths were not much different from the totals of digestive diseases excluding peptic ulcer and appendicitis, and since the great bulk of any transfer of deaths into or out of the cancer group by wrong certification must pass out of or into the " other digestive " group, this means that a strong negative correlation must exist between death rates attributed to the two groups if the large variation in stomach cancer rates arises from diagnostic differences amongst the towns. Indeed, if this were the main cause, doubling the stomach cancer rate (as in Group (*a*) compared with Group (*d*) towns) would involve almost abolishing the rate for other digestive diseases. If there is no appreciable negative correlation the diagnostic factor cannot be of any importance as a cause of the variation. Transfer might also occur between stomach cancer and peptic ulcer as a result of mistaken diagnosis, but the numbers of deaths attributed to the latter group were small in comparison with those attributed to the former, except amongst males aged 45–65. It follows that the transfer of the whole of them to cancer, if that were possible, in certain towns would fail to account for the excess of certified cancer in those towns ; and even large negative correlations between the two causes could not account for the variation in stomach cancer amongst males over 65 or amongst females of either age group.

Table VI shows that for males of both age groups cancer of stomach death rates had no appreciable correlation with either peptic ulcer or other digestive diseases ; for females of both age groups there was a small but statistically insignificant negative correlation with peptic ulcer, and a small just significant positive correlation with other digestive diseases in the country as a whole.

Since none of the factors tested above can account for the differences in Tables I and II, the remaining explanation is that some extrinsic irritant factor is concerned which differed in intensity in the various towns. In order to test further the curious contrasts in cancer of stomach mortality in London boroughs when grouped according to their source of water supply (Registrar-General, 1947), correlation coefficients were calculated between the C.M.R. for cancer of the stomach during 1921–39 and the estimated total hardness of the water supply of the 83 towns during 1911–31, as shown in Tables I and II. It has to be remembered that the location of certain industries has been influenced to some extent in the past by the need for a soft water supply, and that in the north of England much of the industrial population is served by soft water. Of the 42 northern towns 23 had water supplies with less than 7 degrees, or 10 parts per 100,000 of total hardness; their average social class index was about 37 per cent unskilled and partly skilled workers. The 9 northern towns with water supplies of 20 or more parts per 100,000 total hardness had an average social class index about 42 per cent; and the correlation between hardness and social index was significantly positive. In the 41 towns elsewhere there was no correlation between the two indices.

The coefficients of correlation in Table III between cancer of stomach mortality and hardness were positive in the North for males and negative in the South for each sex; and when all the towns are grouped according to an ascending scale of water hardness, it appears that the C.M.R. was low in the groups with moderate hardness and higher in the towns with very soft or very hard water. No explanation is offered for this, but the figures are placed on record because they might at some future time fit in with other facts (Table VIII).

TABLE VIII.—*Comparative Mortality Ratios in Towns of Varying Water Hardness.*

| Hardness per 100,000. | | 0–. | 5–. | 10–. | 15–. | 20–. | 30 and over. | All towns. |
|---|---|---|---|---|---|---|---|---|
| Number of towns | | 15 | 16 | 16 | 13 | 15 | 8 | 83 |
| C.M.R. | Males | 108 | 93 | 110 | 88 | 92 | 102 | 100 |
| | (0–. to 10–. combined) | | 103 | | | | | |
| | Females | 112 | 97 | 110 | 85 | 91 | 100 | 100 |
| | (0–. to 10–. combined) | | 106 | | | | | |

Whatever irritant may be concerned in the production of gastric cancer, the effect of greater intensity of the irritant in one environment than in another might be (1) to increase the proportion of people developing cancer at each age without changing the latent period of development; (2) to shorten the average latent period before cancer appears without changing the proportions of people affected, or (3) to increase the proportion affected and also shorten the latent period. If the irritant is a living organism the average latent period is unlikely to vary, and the first effect would be expected from analogy with other infective processes. If it is a long-continued mechanical or chemical injury to cells the predominant effect to be expected from increased intensity of action would be a shortening of the latent period, without necessarily increasing the proportion of people affected, although that might also occur.

Differences in proportions of people affected in two localities without change in latent period would result in the same relative difference in death rate at each age, provided the numbers dying were small in comparison with the living population. The ratios between death rates in groups of towns and between social groups would not be expected to fall with advancing age after 50 or 60 as in Tables IV and V, and this suggests that the irritant is not an infective organism.

Assuming some form of chemical or mechanical irritant is concerned, the latent period before cancer appears will vary rather widely about a mean value in the manner characteristic of most measurable vital factors ; and if the latent periods of gastric cancers in residents of all towns could be measured they would form a distribution approximating to the " normal " type.

It would be possible to reproduce the observed age-incidence of deaths from gastric cancer on the supposition that the irritant begins to affect a certain proportion, P, of people who are susceptible to it at about 15 years of age, and that at each year of age thereafter an additional proportion P/K become susceptible to it and begin to be affected. If, for example, the mean latent period before death were 20 years, with a standard deviation of 5 and normal distribution, K being a suitable constant, the numbers of deaths at successive quinquennial age periods from 25–29 to 65–69 would give a reasonably good fit to the deaths registered in England and Wales in 1947. A shortening of the latent period in the towns where the irritant was most intense and a lengthening of it in those towns where it was least intense would then result in the ratio between the respective death rates diminishing with advancing age after about 40, as in Table IV. The observed facts appear to be consistent with such an explanation, but a careful study of the mathematics of the assumptions outlined above is needed before definite conclusions can be reached.

### SUMMARY OF CONCLUSIONS.

The death rates from cancer of the stomach in the 83 county boroughs of England and Wales in the period 1921–39 show great differences, which cannot be explained either by chance variations, or by differing accuracy of certification of the cause of death. Standardized mortality tends to be greater in the northern towns, in towns with a low proportion of people of advanced age and in towns with a high proportion of men in unskilled and partly skilled occupations. The contrast between death rates in the towns with high and low mortality diminishes with advancing age after about 45. Towns with a water supply of moderate hardness tended to have lower rates than towns with soft or very hard water. Cancer of the stomach death rates of men were unrelated with those for peptic ulcer or other digestive diseases ; but amongst women there was a small negative correlation with peptic ulcer and a positive correlation with other digestive diseases. The facts seem to be consistent with the hypothesis that gastric cancer depends on an irritant whose latent period is shorter in some towns than in others.

### REFERENCES.

REGISTRAR-GENERAL.—(1931) *Decennial Supplement : Occupational Mortality*.—(1947) *Regional and Local Differences in Cancer Death Rates. Studies in Medical and Population Subjects*, No. 1, p. 25. (1948) *Ibid.*, p. 28.